# Employee psychological well‐being during the COVID‐19 pandemic in Germany: A longitudinal study of demands, resources, and exhaustion

**DOI:** 10.1002/ijop.12743

**Published:** 2021-02-21

**Authors:** Bertolt Meyer, Alexander Zill, Dominik Dilba, Rebecca Gerlach, Susen Schumann

**Affiliations:** ^1^ Department of Psychology Chemnitz University of Technology Chemnitz Germany

**Keywords:** Corona, Exhaustion, Demands, Resources, Lockdown measures

## Abstract

Many governments react to the current coronavirus/COVID‐19 pandemic by restricting daily (work) life. On the basis of theories from occupational health, we propose that the duration of the pandemic, its demands (e.g., having to work from home, closing of childcare facilities, job insecurity, work‐privacy conflicts, privacy‐work conflicts) and personal‐ and job‐related resources (co‐worker social support, job autonomy, partner support and corona self‐efficacy) interact in their effect on employee exhaustion. We test the hypotheses with a three‐wave sample of German employees during the pandemic from April to June 2020 (*N*_*w*1_ = 2900, *N*_*w*12_ = 1237, *N*_*w*123_ = 789). Our findings show a curvilinear effect of pandemic duration on working women's exhaustion. The data also show that the introduction and the easing of lockdown measures affect exhaustion, and that women with children who work from home while childcare is unavailable are especially exhausted. Job autonomy and partner support mitigated some of these effects. In sum, women's psychological health was more strongly affected by the pandemic than men's. We discuss implications for occupational health theories and that interventions targeted at mitigating the psychological consequences of the COVID‐19 pandemic should target women specifically.

The coronavirus disease (COVID‐19) pandemic that started in December 2019 is a worldwide health crisis affecting millions of people (World Health Organization, 2020). To avoid overloading healthcare systems, governments implemented measures, such as lockdowns, closings of institutions and businesses, and social distancing regulations, to flatten the epidemic curve until vaccines or drugs become available (Anderson et al., 2020). These measures radically affect workplaces, the economy (e.g., unemployment, cf. Rudolph et al., 2020), and individuals in their daily work lives. The closure of companies and educational institutions has forced many of those who did not lose their jobs to work from home, where employees have to cope with the multiple demands of balancing family and work, especially when children are present.

The COVID‐19 pandemic thus confronts employees with severe work‐related and private demands. However, over time, high demands and low levels of resources are likely to deplete individual energy reserves, reducing psychological well‐being (Hobfoll, 2001). We thus argue that the COVID‐19 pandemic poses new challenges for employee psychological health that go beyond previous findings in the area of demands and resources (e.g., Bakker et al., 2014; Sonnentag, 2015). For example, many employees face working from home while simultaneously having to care for children and while having to reduce social contacts to close friends and family members. Working from home and social distancing regulations reduce the amount of available social support, which is a core protective factor against psychological health issues (Halbesleben, 2006). In addition to such work‐related changes, the pandemic threatens psychological well‐being in other ways, including fears about one's own health and the well‐being of loved ones, frustration, loneliness and financial losses (Brooks et al., 2020). As the overall research objective, we thus investigate the relationship between work‐related and personal demands and resources during the pandemic and employees' psychological well‐being. We ask how specific personal factors such as gender and having to work from home interact with employees' demands and resources in affecting emotional exhaustion over the course of the pandemic.

By doing so, we hope to contribute to research on occupational health by expanding the perspective of the job‐demands‐resources model (JD‐R, Bakker et al., 2014) to include pandemic‐related demands and (shortage of) resources in the prediction of employees' emotional exhaustion. We also hope that our results can also serve as starting points for interventions in future crises.

We investigate our hypotheses with a three‐wave study of German employees during the COVID‐19 pandemic from April to June 2020. We focus our research on one country because of the differences in restrictions, infection rates, government interventions and support measures, employment policies, health systems, economic preconditions and the societal system between countries.

## PANDEMIC DURATION, DEMANDS AND RESOURCES AND THEIR EFFECT ON EXHAUSTION

The pandemic affects individuals across the globe in multiple ways. Governments reacted to the immediate threats to public health by restricting daily life in multiple ways as mentioned above. Although effective in slowing the spread of the virus, these government measures are psychological stressors in their own right (Rudolph et al., 2020; Van Bavel et al., 2020).

We attempt to capture the pandemic's impact on employee well‐being by focusing on emotional exhaustion, that is, the feeling that one's emotional resources are depleted, because handling excessive stress requires a substantial amount of resources. Next to depersonalization and reduced feelings of personal accomplishment, exhaustion is the core facet of burnout, defined as psychological strain in response to chronic work stress (Maslach, 1993). Exhaustion is however more strongly related to psychological health impairments than the other two facets and also precedes them (Bakker et al., 2014). Exhaustion and burnout are psychological health issues in their own right and are linked to severe consequences such as depression and suicide (see Schermuly & Meyer, 2016, for a review). Whenever research focuses on psychological health in general and psychological exhaustion in particular, researchers typically take study participants' gender into account, as the levels of self‐reported psychological health issues differ between men and women such that, on average, women tend to report higher levels than men (e.g., Houkes et al., 2011; Innstrand et al., 2011; Maslach et al., 2001; Schaufeli & Enzmann, 1998; see Purvanova & Muros, 2010, for a meta‐analysis).

For predicting employee exhaustion during the pandemic, two theories appear especially relevant, because they speak to the interactions of demands and resources and to the associated temporal dynamics: The job‐demands‐resources model (JD‐R, Bakker et al., 2014), and the conservation of resources (COR) theory (Hobfoll, 2001).

According to the former, job demands (e.g., workload), job resources (e.g., social support) and personal resources (e.g., self‐efficacy) are related to motivation (e.g., work engagement) and strain (e.g., exhaustion) and hence, the job performance of working people (Bakker & Demerouti, 2017). Research on the JD‐R shows that demands and resources predict employees' exhaustion (Bakker & Demerouti, 2017; Hatch et al., 2019) such that job resources can substitute resource losses. A meta‐analytic review of longitudinal studies also showed reversed causal effects of burnout on job characteristics (Lesener et al., 2019), which implies that exhausted employees perceive and have more job demands and less job resources in the future (Bakker & Demerouti, 2017). According to Bakker and Demerouti (2017), research in the context of the JD‐R still exhibits some gaps and we hope to address some of these with this study. Specifically, outside the work environment, the JD‐R framework only focuses on personal resources, but not on personal demands, that is, “the requirements that individuals set for their own performance and behavior that force them to invest effort in their work and are therefore associated with physical and psychological costs” (Barbier et al., 2013, p. 751). Furthermore, most studies on the JD‐R do not capture objective demands but solely rely on their perceptions, resulting in common method bias. By taking personal demands and their objective occurrence (in the form of lockdown measures) into account when studying exhaustion in the context of the COVID‐19 pandemic, we thus hope to contribute to research on the JD‐R as well.

Whereas most JD‐R research focused on independent effects of job characteristics on health‐related outcomes, COR theory suggests that demands deplete resources, and that the (temporal) processes associated with resource depletion cause emotional exhaustion (van Woerkom et al., 2016). Specifically, according to COR theory (Hobfoll, 2001, 2010), people's identity consists of personal resources (e.g., self‐efficacy) and social resources (e.g., close relationships) and individuals maintain psychological health by maintaining these resources. Broadly speaking, resources are “things that people value, with an emphasis on objects, states, conditions, and other things” (Halbesleben et al., 2014, p. 1337). COR theory posits that resource losses are more salient than resource gains and that individuals must invest resources to gain resources, to protect themselves from losing resources, and for recovering from losing resources. From these core principles, several corollaries follow: First, individuals with more resources can gain additional resources more easily, and people with few resources are more likely to experience resource losses. Second, initial resource losses lead to future resource losses, and initial gains lead to future resource gains (Halbesleben et al., 2014; Hobfoll, 2010). COR theory therefore has a strong temporal component and implies that resource loss cycles can occur and that these loss cycles can have “accelerating speed” (Hobfoll, 2010, p. 137). According to the COR model, stress and burnout result from a threat to resources, actual resource loss or insufficient resource gains after investing or losing resources (Halbesleben, 2006). In sum, COR theory construes threats to resources, resource losses, and insufficient resource gains as the core antecedents of stress, burnout and exhaustion (Halbesleben, 2006; Hobfoll, 2010).

COR theory is very explicit about what constitutes a resource; Hobfoll (2001) lists 74 exhaustive personal resources. According to Halbesleben et al. (2014), these can be grouped into categories such as social support (e.g., from co‐workers, spouse), objects/conditions (e.g., income and job security), constructive resources (e.g., autonomy, rewards, control), and energies (e.g., recovery experiences, time away from work). According to COR theory, these objective resources (although experienced on a personal and subjective level), are threatened by “[…] objective elements of threat and loss, and common appraisals held jointly by people who share a biology and culture. This places much greater emphasis on objective reality, and greater focus on circumstances where clear stressors are occurring” (Hobfoll, 2010, p. 127). Therefore, COR theory has been successfully employed in the context of psychological health in the aftermath of natural disasters such as hurricanes (see Hobfoll, 2010, for an overview). Given its temporal focus on accumulating resource losses as antecedents of emotional exhaustion, given its prior application to natural disasters, and given its empiric support (see Halbesleben, 2006, for a meta‐analysis), COR theory appears particularly suited for predicting emotional exhaustion during the COVID‐19 pandemic.

Specifically, the COVID‐19 pandemic and its impact on economic and social aspects of life imply losing several resources from multiple categories: Financial challenges for employers induce job insecurity and potentially threaten employees' objective financial resources. Enforced home office and layoffs threaten social support from supervisors and colleagues while social distancing regulations can lead to losing social support from friends and family. Furthermore, the closing of childcare facilities—which we construe as a personal demand in the context of the JD‐R—threatens the energetic resources of individuals caring for children. In sum, this multitude of potential resource losses connected to the COVID‐19 pandemic implies two temporal dynamics: First, the resource losses and the ensuing resource investments for offsetting these losses are likely to induce accelerating loss cycles, leading to an accelerated increase in emotional exhaustion. This increase of exhaustion can only be offset at a later stage, when an easing of restrictions at later stages of the pandemic results in more resources for offsetting the initial losses (e.g., the re‐opening of childcare facilities and businesses). Therefore, over the course of the pandemic, COR theory implies a curvilinear, inverse u‐shaped trajectory of emotional exhaustion. We thus propose:

**Hypothesis 1:** The duration of the COVID‐19 pandemic affects Exhaustion in an inverse‐u‐shaped nonlinear way.

Second, the first corollary of COR—that individuals with fewer resources are likely to experience resource losses—indicates that the severity of the inverse u‐shaped impact of the pandemic on emotional exhaustion differs for people with differing levels of resources at the onset of the pandemic. On the basis of this reasoning, we propose interactions between certain pandemic‐related demands and resources with the duration of the pandemic such that they attenuate or exacerbate the temporal effect. For reasons of parsimony, we selected certain structural and psychological demands and resources that we deem somewhat representative of the set of resources that underlie COR theory. To re‐iterate, COR theory underscores that objective elements that are likely to affect a large set of individuals should be investigated in the context of (threats of) resource losses. We thus start off by investigating the relationship between government and corporate lockdown measures including work from home arrangements and emotional exhaustion. Working through different categories of resources (Halbesleben et al., 2014), we subsequently investigate the role of job insecurity (representing conditional resources), job autonomy and self‐efficacy (representing constructive resources), and co‐worker and partner social support (representing social support).

We construe lockdown measures as a mix of work‐related and non‐work demands. Prior research shows that employees' psychological well‐being varies over time depending on work or nonwork events and that the experiences of high demands increase exhaustion (Sonnentag, 2015). We construe government measures as non‐work events that are likely to deplete individual's resources, which in turn affect exhaustion. In a nutshell, and in line with the above arguments surrounding our proposition of a curvilinear effect on the basis of COR theory, we assume that the extent of exhaustion increases, when restrictions are introduced and decreases, when governments ease restrictions.

**Hypothesis 2:** Pandemic‐related restrictions affect exhaustion such that introducing restrictions increase exhaustion and such that loosening restrictions decrease exhaustion.

In the context of COR theory, social support is a category of resources in its own right (Halbesleben et al., 2014). Among the 74 relevant resources associated with the COR model, several are of a social nature, including “support from co‐workers,” “understanding from my employer/boss,” “help with tasks at home” and “intimacy with spouse or partner.” All of these can have an important impact on individuals' identity and are thus seen as resources in their own light. Accordingly, the positive relationship between social support and psychological health is well‐established (see Halbesleben, 2006, for a meta‐analysis). Given our above arguments proposing that individuals with fewer resources will experience more strain during the pandemic, we propose:

**Hypothesis 3:** Social support, both at work and at home, moderate the curvilinear relationship between pandemic duration and exhaustion such that more support attenuates the relationship and less support strengthens it.

According to COR theory, job security is a central conditional resource (Halbesleben et al., 2014), and individuals are thus likely to invest resources to maintain job security and to fight off threats to their job security. Therefore, on the flipside, job insecurity, “a perceived threat to the continuity and stability of employment as it is currently experienced” (Shoss, 2017, p. 1914), represents a low level of this resource, which—according to the first corollary of COR theory (see above)—makes individuals more prone to suffer other resource losses during the pandemic.

The general relationship between job insecurity and psychological well‐being and burnout is indeed well documented: Meta‐analyses have found consistent relationships between job insecurity and negative psychological health outcomes (Cheng & Chan, 2008; Sverke et al., 2002). Longitudinal studies also point to a causal relationship between job insecurity and burnout (Dekker & Schaufeli, 1995; Hu & Schaufeli, 2011; Norlund et al., 2010) and also between job insecurity and emotional exhaustion (Kinnunen et al., 1999, 2014). Of note, prior research also found stronger relationships between job insecurity and psychological health issues for women (Mäkikangas & Kinnunen, 2003; Mauno & Kinnunen, 1999).

When it comes to pandemic‐related demands, job insecurity is evidently important: The economic decline during the pandemic is likely to increase job insecurity, with the corresponding effect on exhaustion over time. However, in the context of the pandemic, we need to take the temporal dynamic of the situation into account. Above, we hypothesized an inverse u‐shaped relationship between the duration of the COVID‐19 pandemic and emotional exhaustion. Given that COR theory states that individual with fewer resources (i.e., here: individuals with lower levels of job security) are more likely to experience resource losses and consequently burnout, job insecurity is likely to exacerbate the (inverse u‐shaped) effect of pandemic duration on burnout. We thus propose:

**Hypothesis 4:** Job insecurity moderates the curvilinear relationship between pandemic duration and exhaustion such that more job insecurity exacerbates the relationship and less insecurity attenuates it.

We further propose that conflicts between work and privacy are especially important during the pandemic as many people work from home and depend more strongly on their family for social support in the presence of social distancing measures. In general, research on the work‐home interface shows a decrease in well‐being when work and private life are in conflict (Sonnentag, 2015). Indeed, several meta‐analyses (e.g., Amstad et al., 2011; Nohe et al., 2015) show that work interference with family consistently decreases psychological well‐being, even if controlling for reciprocal effects (Nohe et al., 2015). In the context of our temporal framework, we thus hypothesise:

**Hypothesis 5:** Conflicts between work and privacy moderate the curvilinear relationship between pandemic duration and exhaustion such that more conflict strengthens the relationship and less conflict attenuates it.

Next to objective and conditional resources, COR theory construes job autonomy and skill discretion, among other things, as so‐called constructive resources (Halbesleben et al., 2014). Job autonomy, that is, the sense of having the choice to initiate and regulate actions at work, is a central job‐related resource because it allows employees to react to specific work situations in idiosyncratic ways (Spreitzer, 1995). Especially, during a crisis such as the COVID‐19 pandemic that entails abrupt challenges and requires flexible reactions, autonomy can be an important resource for employees' well‐being (Schermuly & Meyer, 2016).

With regard to skill discretion, its subjective counterpart is self‐efficacy, individuals' beliefs in their innate abilities (Bandura, 1997). Prior research distinguishes between general and domain‐specific self‐efficacy (Bandura, 1997) and found that domain‐specific efficacy is especially important for work‐related psychological well‐being (Shoji et al., 2016). We thus posit that pandemic‐specific self‐efficacy, that is, the individuals' optimism to face and cope with the pandemic's demands (based on Shoji et al., 2016), as an important non‐work resource and propose:

**Hypothesis 6:** The constructive resources job autonomy and pandemic‐specific self‐efficacy moderate the curvilinear relationship between pandemic duration and exhaustion such that more resources attenuate the relationship and less support strengthen it.

All of the hypotheses speak to emotional exhaustion as dependent variable. We thus deem it important to note that prior research has often found that women report higher levels of emotional exhaustion than men (e.g., Houkes et al., 2011; Innstrand et al., 2011; Maslach et al., 2001; Schaufeli & Enzmann, 1998; see Purvanova & Muros, 2010, for a meta‐analysis). This is due to working women's additional responsibilities at home and their resulting higher overall workloads (Byron, 2005; Eek & Axmon, 2015; Schaufeli & Enzmann, 1998). We thus investigate whether the effects that we hypothesise above differ between men and women.

## METHOD

### Sample

We collected data in three waves over a period of 3 months from voluntary participants from the general population in a non‐representative way as we describe below. The original dataset consisted of 3862 individuals at the first wave. Our analysis is based on the subset of German participants who work, *n*_*w*1_ = 2900, *n*_*w*12_ = 1237, *n*_*w*12*3*_ = 789. The sample description below refers to those 789 participants who completed all three waves. For the full description of all demographic variables across all waves, see Appendix A1 in the Supporting information (see also Meyer et al., 2021). The majority of participants were female (69.20%, male = 30.04%), with an average age of *M* = 41.94 years (*SD* = 11.38). Our sample consisted of regular employees (83.40%), tenured government officials (7.22%), university students who work (3.93%) and freelancers (3.68%). Participants worked in diverse professional sectors, with 22.94% working in public administration, 20.53% in health and social services, 10.77% at universities and research institutions, 7.73% in the service sector and 31.06% distributed across 25 other sectors (no response: 6.97%). About one‐third (34.35%; no response: 6.34%) provided critical services and infrastructure. The prevalence of working from home declined over time (w_1_:39.92%, w_2_: 33.97%, w_3_: 20.41%). The same was true for a combined on‐site/work from home scenario (21.17%, 18.63% and 17.74%, respectively).

The majority of participants were living with a partner (w_1_: 75.03%) in a shared household (86.15%). The prevalence of partners working from home fluctuated over time (w_1_: 42.91%, w_2_: 47.56%, w_3_: 39.04%). Household size was measured once at wave_1_ (*M* = 2.51, *SD* = 1.19); 40.30% of the participants reported that no children live in their household (no response: 24.08%). Individually, 57.67% reported no pre‐school children (one child: 10.27%, two children: 7.22%, more than two children: 0.89%). Similarly, 51.84% of participants reported no school‐age children (one child: 13.56%, two children: 9.25%, more than two children: 1.02%). Among the 281 participants with either school‐age or pre‐school children, 87.19% reported closures of daycare facilities for their children at wave_1_. At wave_2_, 56.58% reported no change (no response: 21.35%), while 12.46% reported an improvement, whereas 9.61% reported a deteriorating situation. At wave_3_ (no response: 49.82%), 22.78% reported no change, 25.27% an improvement and 2.14% worsening conditions for childcare.

All procedures performed in studies involving human participants were in accordance with the ethical standards of the Institutional Research Board (“Ethikkommission”) of the Chemnitz University of Technology and with the 1964 Helsinki Declaration and its later amendments or comparable ethical standards. The IRB review protocol and decision are available in the OSF repository for the study (Meyer et al., 2021). Informed consent was obtained from all individual adult participants included in the study; children were not allowed to participate.

### Procedure

We obtained approval from our University's IRB and subsequently distributed a link to the first wave online. The link was published on multiple platforms and shared by many organisations and institutions.

We informed participants about our university's privacy and data protection policy—which conforms to European and German data protection regulations—and the aims of the study and participants gave informed consent. Upon finishing the main questionnaire, participants could volunteer for two more survey waves by providing their email address, which we stored independently from the survey data. Each participant had to generate a pseudonym code, which was used throughout the study to match data across the three study waves. Importantly, incoming questionnaires were time‐stamped.

Data collection took place over a period of 3 months (April 3rd to June 30th, 2020) and was organised in three overlapping waves. We started the first wave (study week 1) 2 weeks after the onset of a government‐initiated lockdown in Germany. The second wave commenced 4 weeks later on May 4th 2020 when the lockdown was still in place. The third wave started another 4 weeks later on June 2nd 2020, with lockdown restrictions starting to ease. All three waves were ongoing, that is, individuals could still fill in questionnaires for the first measurement point at the end of June.

### Instruments

*Pandemic‐related restrictions*. We obtained person‐level lockdown restrictions for a given participant on the basis of the participant's time stamp and location (obtained by asking for the first two zipcode digits), with the public German ZPID lockdown dataset (Steinmetz et al., 2020). It includes restrictions like social distancing or wearing face masks for each German federal state. At the time of writing, this dataset only covered March 8th–May 15th, 2020. We thus extended it with publicly available federal and state‐level government regulations. We focused on mandated mask wearing (0: no obligation; 1: mandated mask wearing on public transport or in shops; 2: general mandated mask wearing) and pre‐school/kindergarten availability (0: closed to the general public, only emergency services for employees maintaining critical services or infrastructure; 1: open to the general public—again obtained through publicly available government regulations).

For the following psychological constructs, all questionnaire measures used the same Likert‐style agreement format, ranging from 1 (*not at all*) to 5 (*absolutely*) unless stated otherwise. The survey included additional measures (e.g., presenteeism, selfcare), which were not analysed and reported in this study, but are listed in our data transparency table that includes all items, see Appendix A2 in the supplemental material (see also Meyer et al., 2021).

*Exhaustion*. We assessed exhaustion with the Copenhagen Burnout Inventory's (CBI, Kristensen et al., 2005) subscale Personal Burnout. We adapted the temporal reference of the items to fit our specific context, for example, “Since the [start of the Corona crisis]/[last measurement] I feel emotionally exhausted.” Internal validity was high (6 items, *ω*_total1,2,3_ = .95).

*Autonomy*. We assessed autonomy with three items developed by Spreitzer (1995). An example item is “I have significant autonomy in determining how I do my job.” (*ω*_total1,2,3_ = .84).

*Job Insecurity*. We developed three items to measure pandemic‐specific job insecurity, for example, “Due to the Corona pandemic, my job is at risk.” Internal validity was high (*ω*_total1,2_ = .83, *ω*_total3_ = .82).

*Perceived Social Support*. We assessed two sources of social support, that is, co‐workers and partners. Co‐worker support was assessed with two items and adapted appropriately, for example, “How often do you currently get help and support from your [coworkers] [while working from home]?” (Nübling et al., 2005). Responses ranged from 1 (never/hardly ever) to 5 (always). Averaged across all measurement points, the respective inter‐item correlations are *r*_colleagues,on‐site_ = .63, *r*_colleagues,work from home_ = .66. We further employed the Partner Support Scale (8 items, Straughen et al., 2013), for example, “My partner is someone who understands how I am feeling.” Internal validity was consistently high, with *ω*_total1,2_ = .93, *ω*_total3_ = .92.

*Work‐Privacy/Privacy‐Work Conflict*. We used parts of the Copenhagen Psychological Questionnaire (COPSOQ, Nübling et al., 2005) to assess Work‐Privacy Conflict (5 items) and Privacy‐Work Conflict (5 items, adapted to refer either to work from home or on‐site). Example items are “The demands at work interfere with my private and family life” for work‐privacy conflict and “The demands of my private and family life interfere with [my work]/[working from home]” for privacy‐work‐conflict. Internal validity was high for all scales in all three waves (*ω*_total_ = [.93–.96]).

*Pandemic‐Specific Self‐Efficacy*. We developed four items for Pandemic‐Specific Self‐Efficacy, for exmaple,” I feel like I can contribute something important during this corona pandemic” (*ω*_total1_ = .77, *ω*_total2_ = .73, *ω*_total3_ = .76).

## RESULTS

We conducted all analyses with R Version 4.0.2 (R Core Team, 2020). The analysis script is available from the OSF repository (Meyer et al., 2021). Bivariate correlations of study variables at Wave 1 are given in Table 1. Bivariate correlations among measurement variables across all waves are given in Table S1 in the supplementary material and on the OSF repository. We also conducted an attrition analysis, that is, we compared study variables from participants who only participated during the first study wave with those who participated at all waves (and those who participated at the first two waves with those who participated in all waves), see Table 2. Results show the so‐called healthy worker effect in the sense that there was a tendency for participants with higher levels of resources and psychological well‐being to remain in the study, while initial participants with lower levels of resources participated in the subsequent waves to lesser extents. Thus, the below findings pertaining to emotional exhaustion are likely to be on the conservative side, as individuals with higher levels of exhaustion tended to drop out more frequently. An attrition analysis of all demographic variables is available in Table S2 in the supplemental material.

**Table 1:** Means, standard deviations and correlations with confidence intervals of measurement variables

Variable	N	M	SD	1	2	3	4	5	6	7	8	9	10	11	12	13	14	15	16	17
1. Study week	2607	3.69	2.17																	
2. Gender (0 = female, 1 = male)	2870	0.32	0.47	−.03 [−.07, .00]																
3. Gender (0 = female, 1= diverse)	2870	0.00	0.06	−.02 [−.06, .02]	−.04* [−.07, −.00]															
4. Age	2892	41.48	11.37	.16** [.13, .20]	.06** [.02, .10]	.00 [−.03, .04]														
5. Pre‐school children	2250	0.35	0.67	−.07** [−.11, −.02]	.01 [−.03, .05]	−.01 [−.05, .03]	−.19** [−.23, −.15]													
6. School children	2252	0.52	0.80	.08** [.04, .12]	−.03 [−.08, .01]	−.01 [−.06, .03]	.13** [.09, .17]	−.00 [−.04, .04]												
7. Home office (0 = yes, 1 = no)	2900	0.34	0.47	.06** [.02, .10]	.00 [−.03, .04]	.01 [−.02, .05]	.08** [.04, .11]	−.08** [−.12, −.04]	.01 [−.03, .06]											
8. Home office (0 = yes, 1 = both from home and on site)	2900	0.20	0.40	.04 [−.00, .07]	−.00 [−.04, .03]	−.03 [−.06, .01]	.04* [.00, .08]	−.00 [−.04, .04]	.05* [.01, .09]	−.36** [−.39, −.33]										
9. Mandatory mask wearing (0 = no, 1 = yes)	2176	0.28	0.45	.82** [.81, .83]	−.03 [−.07, .02]	−.03 [−.07, .01]	.11** [.07, .16]	−.03 [−.08, .02]	.08** [.03, .13]	.03 [−.01, .08]	.05* [.00, .09]									
10. Childcare (0 = closed, 1 = open)	2176	0.02	0.14	.42** [.39, .46]	−.02 [−.06, .02]	−.01 [−.05, .04]	−.01 [−.05, .03]	−.02 [−.07, .03]	.03 [−.02, .07]	.03 [−.02, .07]	.00 [−.04, .04]	.23** [.19, .27]								
11. Coworker support	2348	3.44	1.06	−.01 [−.05, .03]	−.01 [−.05, .03]	−.02 [−.06, .02]	−.10** [−.14, −.06]	−.10** [−.14, −.05]	−.07** [−.12, −.03]	−.06** [−.10, −.02]	.02 [−.02, .06]	−.03 [−.08, .01]	.02 [−.03, .06]							
12. Partner support	2025	4.33	0.72	−.00 [−.05, .04]	.08** [.04, .12]	−.01 [−.06, .03]	−.07** [−.11, −.03]	−.06* [−.10, −.01]	−.11** [−.16, −.07]	−.02 [−.06, .02]	.00 [−.04, .05]	.01 [−.04, .06]	−.02 [−.07, .03]	.12** [.08, .17]						
13. Autonomy	2562	4.08	0.83	.01 [−.03, .05]	.08** [.04, .12]	−.00 [−.04, .03]	.08** [.05, .12]	.00 [−.04, .04]	−.01 [−.05, .04]	−.20** [−.24, −.16]	.08** [.04, .11]	.03 [−.01, .07]	−.02 [−.06, .02]	.16** [.12, .19]	.08** [.03, .12]					
14. Job insecurity	2781	1.94	0.92	−.06** [−.10, −.02]	−.01[−.05, .03]	.02 [−.02, .06]	−.04* [−.08, −.01]	.03 [−.01, .07]	.02 [−.02, .06]	−.07** [−.11, −.03]	−.03 [−.07, .00]	−.02 [−.06, .02]	.02 [−.02, .06]	−−.09** [−.13, −.05]	−.12** [−.16, −.07]	−.00 [−.04, .03]				
15. Work privacy conflict	2550	2.39	1.01	−.02 [−.06, .02]	−.03 [−.06, .01]	.03 [−.01, .06]	−.03 [−.07, .01]	.24** [.20, .28]	.15** [.11, .19]	−.03 [−.07, .00]	−.01 [−.05, .03]	.02 [−.02, .06]	.02 [−.02, .06]	−−.23** [−.27, −.20]	−.21** [−.25, −.17]	−.14** [−.18, −.11]	.16** [.12, .20]			
16. Privacy work conflict	2461	2.02	1.05	−.01 [−.05, .03]	−.05** [−.09, −.01]	−.01 [−.05, .03]	−.11** [−.15, −.08]	.44** [.40, .48]	.27** [.23, .32]	−.21** [−.24, −.17]	.09** [.05, .13]	.04* [.00, .09]	.01 [−.03, .05]	−−.20** [−.24, −.16]	−.21** [−.26, −.17]	−.04* [−.08, −.00]	.12** [.08, .16]	.47** [.44, .50]		
17. Corona‐specific self‐efficacy	2640	2.89	0.82	−.02 [−.06, .02]	.11** [.07, .15]	.01 [−.02, .05]	.03 [−.01, .07]	−.14** [−.19, −.10]	−.02 [−.06, .02]	−.00 [−.04, .03]	.00 [−.03, .04]	−.07** [−.11, −.03]	−.01 [−.05, .04]	.18** [.14, .22]	.10** [.06, .15]	.17** [.13, .21]	−.19** [−.22, −.15]	−.20** [−.24, −.16]	−.25** [−.29, −.21]	
18. Exhaustion	2632	2.40	1.09	.07** [.04, .11]	−.14** [−.18, −.11]	−.00 [−.04, .04]	−.08** [−.11, −.04]	.20** [.16, .24]	.10** [.06, .15]	−.01 [−.05, .03]	−.04* [−.08, −.00]	.08** [.04, .12]	.05* [.00, .09]	−.21** [−.25, −.17]	−.22** [−.26, −.18]	−.17** [−.20, −.13]	.17** [.14, .21]	.44** [.41, .47]	.39** [.35, .42]	−.49** [−.52, −.46]

*Note. M* and *SD* are used to represent mean and standard deviation, respectively. Values in square brackets indicate the 95% confidence interval for each correlation. The confidence interval is a plausible range of population correlations that could have caused the sample correlation.

^*^ *p* < .05.

^**^ *p* < .01.

**Table 2:** Attrition analysis: *N*s, means and standard deviations for the whole sample, the final sample and drop‐outs in between

	Whole sample (2900)			Only completed Wave 1, then dropped out (1663)			Completed Waves 1 and 2, then dropped out (447)			Completed all three Waves (789)		
Variables	N	M	SD	N	M	SD	N	M	SD	N	M	SD
Age (Wave 1)	2892	41.48	11.37	1657	41.23^a^	11.53	447	41.60^a^	10.76	787	41.94^a^	11.38
Co‐worker support (Wave 1)	2348	3.44	1.06	1239	3.39^a^	1.08	393	3.44^ab^	1.04	715	3.51^b^	1.06
Co‐worker support (Wave 2)							318	3.52^a^	0.98	702	3.56^a^	0.99
Partner support (Wave 1)	2025	4.33	0.72	1074	4.30^a^	0.75	359	4.33^a^	0.64	591	4.36^a^	0.70
Partner support (Wave 2)							285	4.28^a^	0.70	564	4.33^a^	0.68
Autonomy (Wave 1)	2562	4.08	0.83	1378	4.02^a^	0.85	423	4.14^b^	0.80	760	4.14^b^	0.79
Job insecurity (Wave 1)	2781	1.94	0.92	1551	1.96^a^	0.92	445	1.94^a^	0.90	784	1.89^a^	0.92
Job insecurity (Wave 2)							379	1.91^a^	0.90	754	1.81^a^	0.84
Work‐privacy conflict (Wave 1)	2550	2.39	1.01	1370	2.43^a^	1.00	422	2.42^a^	1.09	757	2.29^b^	0.98
Work‐privacy conflict (Wave 2)							342	2.36^a^	1.06	730	2.26^a^	0.97
Privacy‐work conflict (Wave 1)	2461	2.02	1.05	1310	2.03^ab^	1.03	409	2.12^a^	1.15	741	1.94^b^	1.03
Privacy‐work conflict (Wave 2)							331	2.08^a^	1.06	719	1.92^b^	0.97
Corona‐specific self‐efficacy (Wave 1)	2640	2.89	0.82	1406	2.86^a^	0.81	445	2.92^a^	0.81	788	2.2^a^	0.85
Corona‐specific self‐efficacy (Wave 2)							355	2.95^a^	0.82	748	2.93^a^	0.78
Emotional exhaustion (Wave 1)	2632	2.40	1.09	1399	2.45^a^	1.09	446	2.45^a^	1.12	786	2.28^b^	1.05
Emotional exhaustion (Wave 2)							354	2.53^a^	1.07	750	2.42^a^	1.05

*Note*. For the main analyses with the final dataset, gender was an important control variable. However, there was only one person who identified as non‐binary, so they were removed for the final analyses. This results in *N* = 789 instead of *N* = 790 for the final dataset.

The means of the three different subsamples were compared using linear models and appropriate dummy coding (W1 vs. W1–W2 and W1–W3; W1–W2 vs. W1 and W1–W3). No controls for multiple comparisons were applied to highlight potential differences between the subsamples. Samples with the same superscript letter are not significantly different from each other at α = .05.

### Analysis strategy

All hypotheses posit an effect of the duration of the pandemic (i.e., of time) on exhaustion. To test it longitudinally, we used the subsample of 789 individuals who participated at all three measurement waves and who stated that they currently work. In this data, repeated measures of exhaustion exhibited within‐person non‐independence, ICC(1) = 0.68, *F*(789, 1484) = 7.19, *p* < .001, thus, requiring mixed models (i.e., multilevel modelling or random coefficient modelling), which we conducted with the lme4 package (Bates et al., 2014). The repeated measures of exhaustion were also fairly homogeneous within participants, ICC(2) = 0.86.

Given that the study waves were relatively long (wave 1 includes measures spaced up to 12 weeks apart), the variable measurement wave, coded as 1, 2 and 3, was unsuitable for operationalising the duration of the pandemic. We thus used study week (coded as 1–13) from the time stamps of the online questionnaire as the time variable, with three values of study week nested in participants. To model the proposed quadratic inverse u‐shaped effect of time, we included the linear effect of study week and the quadratic effect of study week in the models with polychoric contrasts (Bliese, 2016), which results in a linear and a quadratic time variable that are uncorrelated. This allows a clear separation of linear and quadratic effects. Indeed, a random‐intercept model regressing exhaustion on the quadratic and linear effect of study week fitted to the data better, BIC = 5608.43, than a model that only included the linear effect of time as a predictor, BIC = 5614.03. In the model with the linear and quadratic effects of study week on exhaustion, only the quadratic fixed effect reached significance, *b* = −2.21, 95% CI = [−3.43; −0.98], but not the linear one, *b* = 0.52, 95% CI = [−0.69; 1.73]. To identify the structure of the random effects for this model, we followed the recommendations by Bliese (2016) and found that, in comparison to the random‐intercept model, a random‐intercept‐ and slope model with a random intercept and a random slope for the quadratic effect of study week on exhaustion fitted the data best, Δχ_(2)_^2^ = 12.25, *p* < .01. In this baseline model, all variance components' confidence intervals excluded 0.

### Hypotheses tests

Hypothesis 1 proposed that the duration of the pandemic, that is, study week, affects exhaustion in an inverse u‐shaped way. To test it, we added the control variables age and gender to the previous model, see Model 1a in Table 3. It revealed a significant quadratic effect of study week on exhaustion, *b* = −2.17, 95% CI = [−3.44, −0.91]. Given that it also revealed a significant effect of gender such that men report lower levels of exhaustion than women and given the prevalence of gender effects in exhaustion (e.g., Halbesleben, 2006), we further investigated whether the quadratic effect of time was also gender‐specific by adding a corresponding interaction, see Model 1b. Indeed, the quadratic effect of study week was only present for women (who are represented by the model intercept), but not for men, as visible in the non‐significant interaction between the quadratic effect of study week and being male. Therefore, Hypothesis 1 was only partially supported.

**Table 3:** Mixed models regressing repeated within‐person measures of exhaustion on study variables (see text, *N*_*obs*_ = 2262, *N* = 789)

	Model 1a			Model 1b			Model 2a			Model 2b		
Term	b	95% CI	t	b	95% CI	t	b	95% CI	t	b	95% CI	t
Within‐person fixed effects												
Intercept	2.42	[2.35, 2.50]	61.56	2.43	[2.35, 2.50]	61.48	2.28	[2.09, 2.47]	23.81	2.22	[2.03, 2.42]	21.90
Study week (linear)	0.68	[−0.52, 1.88]	1.11	1.17	[−0.26, 2.61]	1.61	−0.75	[−4.64, 3.14]	−0.38	−0.72	[−4.61, 3.17]	−0.36
Study week (quadratic)	**−2.17**	**[−3.44, −0.91]**	**−3.39**	**−2.61**	**[−4.12, −1.11]**	**−3.41**	0.07	[−2.69, 2.84]	0.05	0.37	[−2.49, 3.26]	0.25
Home office (0 = yes, 1 = no)							**−0.17**	**[−0.30, −0.04]**	**−2.57**	−0.15	[−0.31, 0.01]	−1.90
Home office (0 = yes, 1 = other)							−0.05	[−0.24, 0.13]	−0.55	0.00	[−0.23, 0.22]	−0.04
Home office (0 = yes, 1 = both from home and on site)							**−0.23**	**[−0.35, −0.11]**	**−3.66**	**−0.23**	**[−0.38, −0.08]**	**−3.04**
Mandatory mask wearing							**0.23**	**[0.06, 0.41]**	**2.59**	**0.26**	**[0.07, 0.45]**	**2.74**
Childcare (0 = closed, 1 = open)							−0.08	[−0.21, 0.06]	−1.12	−0.08	[−0.21, 0.06]	−1.11
Between‐person fixed effects												
Gender (0 = female, 1 = male)	**−0.24**	**[−0.38, −0.10]**	**−3.36**	**−0.25**	**[−0.39, −0.11]**	**−3.46**	**−0.32**	**[−0.49, −0.15]**	**−3.73**	−0.12	[−0.40, 0.16]	−0.83
Age (z‐transformed)	**−0.10**	**[−0.17, −0.04]**	**−3.16**	**−0.10**	**[−0.17, −0.04]**	**−3.15**	**−0.11**	**[−0.19, −0.03]**	**−2.68**	**−0.11**	**[−0.19, −0.03]**	**−2.70**
Pre‐school children in household							**0.26**	**[0.15, 0.37]**	**4.52**	**0.28**	**[0.15, 0.41]**	**4.16**
School children in household							**0.12**	**[0.01, 0.22]**	**2.19**	**0.16**	**[0.03, 0.28]**	**2.45**
Cross‐level interactions												
Study week (linear) × gender male				−1.66	[−4.27, 0.95]	−1.25						
Study week (quadratic) × gender male				1.50	[−1.26, 4.25]	1.07	2.96	[−0.47, 6.38]	1.69	1.66	[−2.53, 5.82]	0.78
Pre‐school children × childcare open							**−0.13**	**[−0.23, −0.03]**	**−2.57**	**−0.13**	**[−0.25, −0.02]**	**−2.21**
Gender male × WfH: None										−0.06	[−0.33, 0.21]	−0.46
Gender male × WfH: Other										−0.17	[−0.57, 0.23]	−0.82
Gender male × WfH: Some										0.02	[−0.24, 0.28]	0.12
Gender male × mask wearing										−0.12	[−0.31, 0.07]	−1.20
Gender male × School children										−0.13	[−0.35, 0.10]	−1.09
Gender male × pre‐school children										−0.09	[−0.33, 0.15]	−0.71
Gender male × pre‐school‐children × Childcare										0.02	[−0.18, 0.22]	0.19
Variance components												
Random intercept σ_b1_^2^	0.73	[0.65, 0.81]		0.73	[0.65, 0.81]		0.66	[0.56, 0.76]		0.66	[0.56, 0.75]	
Random slope: study week (quadratic) σ_b2_^2^	20.89	[1.23, 52.02]		21.01	[1.05, 51.73]		21.28	[0.96, 60.2]		22.65	[0.99, 61.91]	
Intercept‐slope covariance σ_b3_^2^	−1.86	[−3.77, −0.67]		−1.84	[−3.79, −0.66]		−2.02	[−3.75, −0.59]		−1.99	[−3.78, −0.57]	
Within‐person variance σ^2^	0.33	[0.30, 0.36]		0.33	[0.30, 0.36]		0.32	[0.29, 0.36]		0.32	[0.29, 0.36]	
												
Observations	2249.00			2249.00			1426.00			1426.00		
Deviance (−2LogLik)	5486.85			5479.27			3437.92			3450.04		
AIC	5504.85			5501.27			3473.92			3500.04		
BIC	5556.31			5564.17			3568.65			3631.60		
Overall pseudo‐*R* ^*2*^_*m*_	0.02			0.03			0.08			0.08		

Hypothesis 2 proposed that introducing COVID‐19‐related restrictions increases exhaustion and that loosening restrictions decreases exhaustion. To test it, we included three restrictions as within‐person time‐varying variables: Work from home arrangements (coded as full‐time work from home, which also represents the intercept, a combination of work from home and on site, and regular work on‐site), mandatory mask wearing, and the closing and re‐opening of child‐care facilities while controlling for the number of pre‐school children and school children in the household. We also added an interaction between the re‐opening of childcare and the number of pre‐school children to the model, see Model 2a in Table 3.

The number of pre‐school and school children were both significantly positively associated with exhaustion. Working from home (represented by the model intercept) was also associated with higher levels of exhaustion, as visible in the lower levels of exhaustion for individuals who only work at home part time or those who work on‐site. The introduction of mandatory mask wearing was also associated with increased exhaustion. The number of pre‐school children and the re‐opening of childcare facilities interacted such that the re‐opening reduced exhaustion if the household contained at least one pre‐school child.

**Figure 1:**
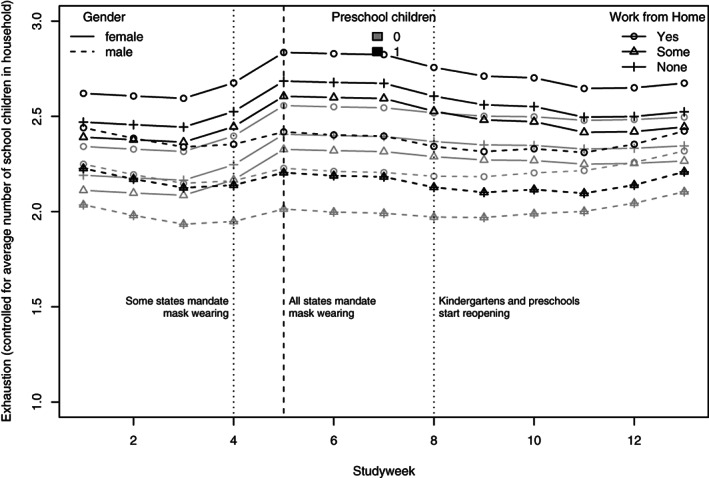
Exhaustion as a function of gender, preschool children, work from home, and the lockdown measures childcare closure/re‐opening and mask wearing (model predictions).

Due to the fact that the previous model revealed a gender‐specificity of the longitudinal effect, we fitted a second model that added interactions with gender to all additional model predictors, see Model 2b. It revealed that all the effects that we found in relation to Hypothesis 2 are present for women only. To facilitate the interpretation of the model, we plot its predictions for different genders, number of children and working arrangements of the study weeks, see Figure 1. In this plot, the introduction of mandatory mask wearing and the re‐opening of childcare centres correspond to the observed occurrences of these measures.

The model and plot reveal that women's exhaustion increases at the onset of mandatory mask wearing. For women with pre‐school children, it decreases when kindergartens reopen. Men's exhaustion is hardly affected, and men in households without children who do not have to work from home report the lowest levels of exhaustion. We interpret the overall pattern of these results as partially supporting Hypothesis 2.

**Figure 2:**
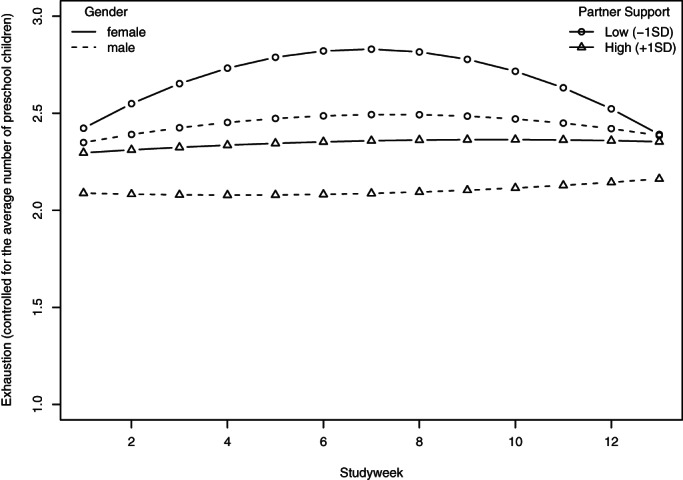
Exhaustion as a function of gender and partner support (model predictions).

Hypothesis 3 proposed that social support, both at work and at home, moderate the curvilinear relationship between pandemic duration and exhaustion such that more support attenuates the relationship and less support strengthens it. For testing this and the following hypotheses, we first fit a baseline model containing the linear and quadratic effect of study week, the interaction between the quadratic effect of study week and gender, and the main effects of the number of children and work from home situation. To this baseline model, we add the main effects of co‐worker and partner social support, see Model 3a in Table 4, and subsequently add interactions between both forms of social support with the quadratic effect of study week and gender, see Model 3b in Table 4. Both models show a significant main effect of co‐worker social support and of partner support on exhaustion that was only present for women. The interaction of partner social support and the quadratic effect of study week turned out to be significant, *b* = 2.15, 95% CI = [0.39; 3.90], but this was not the case for co‐worker social support. To interpret the significant interaction of partner social support with study week, we plotted it, see Figure 2. In line with the hypothesis, emotional exhaustion of women who reported low levels of partner support exhibited a visible inverse u‐shape, while this was not the case for women who reported high levels of support and men in general. The data thus supported Hypothesis 3 in a partial way, that is, only with regard to partner social support (but not with regard to co‐worker support) and only for women.

**Table 4:** Mixed models regressing repeated within‐person measures of exhaustion on study variables (see text, *N*_*obs*_ = 2262, *N* = 789)

	Model 3a			Model 3b			Model 4a			Model 4b		
Term	b	95% CI	t	b	95% CI	t	b	95% CI	t	b	95% CI	t
Within‐person fixed effects												
Intercept (female, no kids, working from home)	2.53	[2.41, 2.64]	43.23	2.52	[2.41, 2.64]	43.10	2.52	[2.41, 2.63]	43.56	2.52	[2.41, 2.63]	43.60
Study week (linear)	0.93	[−0.73, 2.60]	1.09	0.85	[−0.82, 2.52]	0.99	1.61	[−0.08, 3.30]	1.86	1.48	[−0.22, 3.18]	1.70
Study week (quadratic)	**−2.84**	**[−4.65, −1.01]**	**−3.06**	**−2.63**	**[−4.44, −0.80]**	**−2.82**	**−3.03**	**[−4.84, −1.21]**	**−3.28**	**−2.90**	**[−4.72, −1.08]**	**−3.12**
Home office (0 = yes, 1 = no)	**−0.19**	**[−0.31, −0.06]**	**−2.86**	**−0.18**	**[−0.31, −0.05]**	**−2.75**	**−0.17**	**[−0.29, −0.04]**	**−2.58**	**−0.17**	**[−0.29, −0.04]**	**−2.59**
Home office (0 = yes, 1 = other)	0.02	[−0.19, 0.23]	0.20	0.03	[−0.18, 0.23]	0.24	0.01	[−0.20, 0.22]	0.12	0.01	[−0.20, 0.22]	0.08
Home office (0 = yes, 1 = both from home and on site)	**−0.27**	**[−0.39, −0.15]**	**−4.28**	**−0.27**	**[−0.39, −0.14]**	**−4.22**	**−0.25**	**[−0.37, −0.13]**	**−3.98**	**−0.25**	**[−0.37, −0.13]**	**−3.95**
Co‐worker social support (z‐Transformed)	**−0.12**	**[−0.17, −0.06]**	**−4.40**	**−0.14**	**[−0.20, −0.08]**	**−4.43**	**−0.11**	**[−0.16, −0.06]**	**−4.27**	**−0.11**	**[−0.16, −0.06]**	**−4.25**
Partner support (z‐transformed)	**−0.17**	**[−0.22, −0.11]**	**−5.88**	**−0.17**	**[−0.23, −0.10]**	**−5.16**	**−0.16**	**[−0.22, −0.11]**	**−5.68**	**−0.16**	**[−0.22, −0.11]**	**−5.73**
Job insecurity (z‐Transformed)							**0.13**	**[0.07, 0.19]**	**4.36**	**0.11**	**[0.03, 0.18]**	**2.89**
Within‐person fixed effects												
Study week (quadratic) × co‐worker social support				−0.54	[−2.35, 1.27]	−0.59						
Study week (quadratic) × partner support				**2.15**	**[0.39, 3.90]**	**2.40**						
Between‐person fixed effects												
Gender (0 = female, 1 = male)	**−0.25**	**[−0.41, −0.10]**	**−3.19**	**−0.25**	**[−0.40, −0.09]**	**−3.08**	**−0.25**	**[−0.41, −0.10]**	**−3.23**	**−0.25**	**[−0.40, −0.10]**	**−3.18**
Age (z‐transformed)	**−0.15**	**[−0.23, −0.07]**	**−3.74**	**−0.15**	**[−0.23, −0.07]**	**−3.72**	**−0.14**	**[−0.22, −0.06]**	**−3.58**	**−0.14**	**[−0.22, −0.07]**	**−3.62**
Pre‐school children in household	**0.11**	**[0.04, 0.18]**	**3.10**	**0.11**	**[0.04, 0.18]**	**3.02**	**0.12**	**[0.05, 0.19]**	**3.26**	**0.12**	**[0.05, 0.19]**	**3.29**
School children in household	**0.09**	**[0.02, 0.16]**	**2.40**	**0.09**	**[0.02, 0.16]**	**2.38**	**0.08**	**[0.01, 0.15]**	**2.32**	**0.08**	**[0.01, 0.15]**	**2.27**
Cross‐level interactions												
Study week (quadratic) × gender male	2.39	[−1.01, 5.78]	1.38	2.13	[−1.29, 5.54]	1.22	2.65	[−0.74, 6.03]	1.53	2.58	[−0.81, 5.95]	1.49
Gender male × co‐worker social support				0.09	[−0.02, 0.21]	1.62						
Gender male × partner support				−0.06	[−0.20, 0.08]	−0.82						
Study week (quadratic) × gender male × co‐worker social support				0.39	[−3.59, 4.36]	0.19						
Study week (quadratic) × gender male × partner support				−1.03	[−4.76, 2.71]	−0.54						
Study week (quadratic) × job insecurity										−0.84	[−2.76, 1.07]	−0.86
Gender male × job insecurity										0.09	[−0.03, 0.22]	1.48
Study week (quadratic) × gender male × job insecurity										−1.04	[−4.62, 2.54]	−0.57
Gender male × partner support												
Random intercept σ_b1_^2^	0.56	[0.47, 0.64]		0.55	[0.47, 0.63]		0.53	[0.45, 0.61]		0.53	[0.45, 0.61]	
Random slope: study week (quadratic) σ_b2_^2^	8.05	[1.00, 19.65]		6.58	[0.69, 18.55]		6.39	[0.60, 17.91]		6.03	[0.55, 17.56]	
Intercept‐slope covariance σ_b3_^2^	−2.02	[−2.13, −0.61]		−1.90	[−1.93, −0.53]		−1.82	[−1.86, −0.46]		−1.79	[−1.81, −0.46]	
Within‐person variance σ^2^	0.33	[0.30, 0.36]		0.33	[0.30, 0.36]		0.33	[0.30, 0.36]		0.33	[0.30, 0.36]	
Observations	1421.00			1421.00			1417.00			1417.00		
Deviance (−2LogLik)	3381.55			3370.03			3356.38			3350.92		
AIC	3415.55			3416.03			3392.38			3392.92		
BIC	3504.96			3537.00			3486.99			3503.30		
Overall pseudo‐*R* ^*2*^_*m*_	0.13			0.14			0.15			0.16		

Hypothesis 4 postulated that Job insecurity moderates the effect of time on exhaustion. We thus added the main effect of job insecurity to the model while controlling for social support, see Model 4a in Table 4, and an interaction between job insecurity and the quadratic effect of study week, see Model 4b in Table 4. Results revealed a significant negative main effect of job insecurity on exhaustion, but the interaction of the quadratic effect of study week, gender and job insecurity was not significant, rejecting Hypothesis 4.

Hypothesis 5 stated that conflicts between work and privacy moderate the curvilinear relationship between pandemic duration and exhaustion. To test it, we added work‐privacy conflict and privacy‐work conflict to the model in a first step, see Model 5a in Table 5, and the according interaction with the quadratic effect of study week, see Model 5b. Both types of conflict exhibited a strong significant positive association with exhaustion, but no interaction with the duration of the pandemic. The data thus refuted the hypothesis. However, we found a significant interaction between gender and work‐privacy conflict such that men experienced lower levels of exhaustion under high levels of work‐privacy conflict in comparison to women, *b* = −0.19, 95% CI = [−0.31; −0.07].

**Table 5:** Mixed models regressing repeated within‐person measures of exhaustion on study variables (see text, *N*_*obs*_ = 2262, *N* = 789)

	Model 5a		Model 5b				Model 6a			Model 6b		
Term	b	95% CI	t	b	95% CI	t	b	95% CI	t	b	95% CI	t
Within‐person fixed effects												
Intercept	2.45	[2.35, 2.56]	47.06	2.45	[2.35, 2.55]	47.23	2.44	[2.34, 2.53]	50.09	2.44	[2.34, 2.53]	50.16
Study week (linear)	**1.90**	**[0.34, 3.46]**	**2.38**	**2.27**	**[0.69, 3.85]**	**2.80**	**2.94**	**[1.41, 4.48]**	**3.75**	**2.91**	**[1.38, 4.44]**	**3.71**
Study week (quadratic)	**−2.25**	**[−3.98, −0.52]**	**−2.54**	**−2.24**	**[−3.98, −0.48]**	**−2.49**	**−2.31**	**[−3.99, −0.61]**	**−2.66**	**−2.30**	**[−4.01, −0.58]**	**−2.59**
Home office (0 = yes, 1 = no)	−0.09	[−0.21, 0.02]	−1.56	−0.08	[−0.20, 0.03]	−1.41	−0.11	[−0.22, 0.00]	−1.91	−0.11	[−0.22, 0.00]	−1.98
Home office (0 = yes, 1 = other)	0.07	[−0.12, 0.26]	0.69	0.08	[−0.12, 0.27]	0.77	0.07	[−0.11, 0.26]	0.79	0.07	[−0.12, 0.25]	0.72
Home office (0 = yes, 1 = both from home and on site)	**−0.22**	**[−0.33, −0.11]**	**−3.77**	**−0.20**	**[−0.32, −0.09]**	**−3.48**	**−0.20**	**[−0.31, −0.09]**	**−3.55**	**−0.20**	**[−0.31, −0.10]**	**−3.68**
Co‐worker social support (z‐transformed)	**−0.06**	**[−0.10, −0.01]**	**−2.34**	**−0.06**	**[−0.10, −0.01]**	**−2.29**	−0.03	[−0.07, 0.02]	−1.14	−0.02	[−0.07, 0.02]	−1.00
Partner support (z‐transformed)	**−0.10**	**[−0.15, −0.05]**	**−3.83**	**−0.09**	**[−0.14, −0.04]**	**−3.65**	−0.08	**[−0.13, −0.03]**	**−3.36**	**−0.08**	**[−0.13, −0.03]**	**−3.24**
Job insecurity (z‐transformed)	**0.08**	**[0.02, 0.13]**	**2.81**	**0.08**	**[0.03, 0.13]**	**2.88**	0.04	[−0.01, 0.09]	1.56	0.04	[−0.01, 0.09]	1.55
Privacy‐work‐conflict (z‐transformed)	**0.17**	**[0.12, 0.23]**	**6.02**	**0.15**	**[0.09, 0.22]**	**4.59**	0.14	**[0.08, 0.19]**	**4.96**	**0.14**	**[0.09, 0.19]**	**5.00**
Work‐privacy‐conflict (z‐transformed)	**0.36**	**[0.30, 0.41]**	**12.88**	**0.41**	**[0.35, 0.47]**	**12.49**	0.31	**[0.25, 0.36]**	**11.46**	**0.31**	**[0.26, 0.36]**	**11.54**
Corona‐specific self‐efficacy (z‐transformed)							−0.29	**[−0.33, −0.24]**	**−11.44**	**−0.31**	**[−0.37, −0.25]**	**−10.59**
Within‐person fixed effects												
Study week (quadratic) × privacy‐work‐conflict				0.46	[−1.39, 2.32]	0.49						
Study week (quadratic) × work‐privacy‐conflict				−1.54	[−3.43, 0.34]	−1.59						
Study week (quadratic) × autonomy										**1.70**	**[0.13, 3.29]**	**2.10**
Study week (quadratic) × corona‐specific self‐efficacy										−0.63	[−2.46, 1.21]	−0.67
Between‐person fixed effects												
Gender (0 = female, 1 = male)	**−0.26**	**[−0.40, −0.13]**	**−3.86**	**−0.27**	**[−0.40, −0.13]**	**−3.91**	**−0.17**	**[−0.29, −0.05]**	**−2.71**	**−0.19**	**[−0.32, −0.07]**	**−3.01**
Age (z‐transformed)	**−0.09**	**[−0.16, −0.02]**	**−2.52**	**−0.09**	**[−0.16, −0.02]**	**−2.61**	−0.06	[−0.12, 0.00]	−1.84	−0.06	[−0.12, 0.00]	−1.90
Pre‐school children in household	−0.01	[−0.08, 0.05]	−0.41	−0.01	[−0.07, 0.05]	−0.26	−0.03	[−0.09, 0.03]	−0.99	−0.03	[−0.09, 0.03]	−1.04
School children in household	−0.02	[−0.08, 0.05]	−0.53	−0.02	[−0.08, 0.04]	−0.60	−0.01	[−0.07, 0.05]	−0.33	−0.01	[−0.07, 0.05]	−0.30
Autonomy (z‐transformed)							**−0.10**	**[−0.16, −0.05]**	**−3.50**	**−0.14**	**[−0.20, −0.07]**	**−3.92**
Cross‐level interactions												
Study week (quadratic) × gender male	1.61	[−1.62, 4.85]	0.98	1.63	[−1.58, 4.83]	0.99	2.50	[−0.65, 5.65]	1.55	1.94	[−1.30, 5.16]	1.17
Gender male × privacy‐work‐conflict				0.04	[−0.07, 0.16]	0.73						
Study week (quadratic) × gender male × privacy‐work‐conflict				0.28	[−3.46, 4.02]	0.15						
Gender male × work‐privacy‐conflict				**−0.19**	**[−0.31, −0.07]**	**−3.14**						
Study week (quadratic) × gender male × work‐privacy‐conflict				−0.51	[−4.60, 3.59]	−0.24						
Gender male × autonomy										0.08	[−0.05, 0.20]	1.21
Study week (quadratic) × gender male × autonomy										−1.01	[−4.30, 2.24]	−0.60
Gender male × corona‐specific self‐efficacy										0.09	[−0.01, 0.19]	1.75
Study week (quadratic) × gender male × corona‐specific self‐efficacy										1.88	[−1.55, 5.33]	1.07
Variance components												
Random intercept σ_b1_^2^	.38	[0.32, 0.44]		0.38	[0.32, 0.44]		0.30	[0.25, 0.35]		0.30	[0.25, 0.35]	
Random slope: study week (quadratic) σ_b2_^2^	11.00	[0.35, 43.73]		8.40	[0.29, 39.02]		9.46	[0.11, 40.76]		8.88	[0.26, 50.22]	
Intercept‐slope covariance σ_b3_^2^	−1.35	[−2.05, −0.32]		−1.32	[−1.79, 0.06]		−1.06	[−1.69, 0.04]		−1.11	[−1.64, −0.28]	
Within‐person variance σ^2^	0.29	[0.26, 0.32]		0.29	[0.26, 0.32]		0.28	[0.24,0.30]		0.27	[0.24, 0.30]	
Observations	1416.00			1416.00			1382.00			1382.00		
Deviance (−2LogLik)	3111.45			3096.78			2897.35			2885.80		
AIC	3151.45			3148.78			2941.35			2941.80		
BIC	3256.56			3285.42			3056.44			3088.27		
Overall pseudo‐*R* ^*2*^_*m*_	0.33			0.34			0.42			0.43		

**Figure 3:**
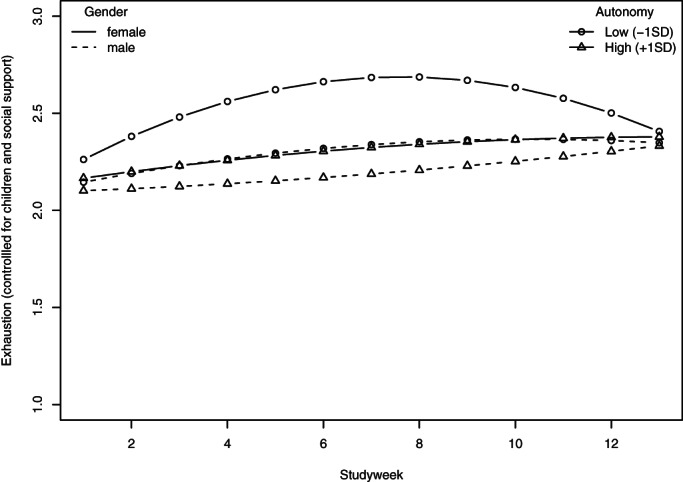
Exhaustion as a function of gender and job autonomy (model predictions).

Finally, Hypothesis 6 stipulated that job autonomy and pandemic‐specific self‐efficacy moderate the curvilinear relationship between pandemic duration and exhaustion. To test it, we added the two corresponding variables to the model, see Models 6a and 6b in Table 5. In partial support of the hypothesis, the analysis revealed a significant interaction between job autonomy and the quadratic effect of study week for women, see Figure 3: The emotional exhaustion of women with relatively low levels of job autonomy rose towards the middle of the study period, while this was not the case for women with high levels of job autonomy or men.

## DISCUSSION

Our study investigated the effects of individual demands, job demands and pandemic‐related demands and resources on employee exhaustion during the COVID‐19 pandemic in Germany. In general, our findings show that all three areas affect employees' exhaustion, but these effects are almost entirely exclusive for women. Women's exhaustion exhibits an inverse u‐shape as a function of government lockdown measures such that women who have to work from home experience most exhaustion during strict lockdown measures such as the closing of childcare facilities, social distancing and mask wearing. Caring for pre‐school children, low levels of job autonomy, and low levels of partner support further exacerbate this gender‐specific increase in exhaustion. On the contrary, in our sample, the pandemic had small effects on the exhaustion of men working from home and/or not taking care for children. These findings echo other findings pertaining to gender differences in psychological health during the current pandemic (Rudolph et al., 2020). Prior research already found that unequal distributions of household and childcare duties are responsible for higher levels of emotional exhaustion among women in comparison to men (Eek & Axmon, 2015). We thus interpret our findings such that the pandemic exacerbated this unequal distribution of duties between men and women, likely because women lost resources that are more crucial to fulfilling multiple responsibilities when childcare facilities closed and when social contacts were cut. If this is true, our findings illustrate that an unequal distribution of family and household duties was present in our sample during the pandemic in Germany and that women therefore had fewer resources to deal with the psychological consequences of the pandemic and therefore suffered resource losses to a stronger extent than men.

Independent from the temporal development of exhaustion during the COVID‐19 pandemic, we found significant main effects for government measures as well as work‐related and personal issues on emotional exhaustion. Pandemic‐specific self‐efficacy and work‐privacy conflict exhibit strong relationships with employee exhaustion.

### Theoretical implications

The findings deepen our understanding of the relation of demands and resources with exhaustion during a crisis above and beyond the work context of the job‐demands‐resources model (JD‐R, Bakker et al., 2014). According to Bakker and Demerouti (2017), one unresolved issue of the JD‐R is its neglect for personal demands outside the immediate job context. Our findings show that such personal demands (i.e., low levels of partner support and potentially unequal distribution of household and childcare duties) can cause emotional exhaustion among working women and that job resources such as job autonomy can offset these personal demands. Therefore, extending the JD‐R to include personal demands alongside job demands appears necessary in the light of our findings. Furthermore, objective societal demands such as lockdown measures are difficult to conceptualise within the JD‐R, as these are neither personal nor job‐related demands. Finally, our findings also show that objective events such as the introduction of and easing of lockdown measures have a notable effect on employees' emotional exhaustion (at least for certain groups), thereby demonstrating that the JD‐R operates outside the confinement of common source bias. In sum, with regard to the JD‐R, our findings highlight the interaction of work‐related issues, personal issues and the broader societal context. Therefore, organisational and occupational health research needs to strengthen approaches conceptualising employees' situation from a multi‐level perspective (e.g., Sonnentag, 2015), where individuals' immediate social contexts such as the workplace or family interact with broader societal developments. This is especially relevant against the backdrop of our finding of significant impacts of government measures (e.g., mask wearing, kindergartens re‐opening) on exhaustion.

Our findings also speak to the usefulness of COR theory (Hobfoll, 2010): In line with its predictions, individuals with fewer resources (less support, less autonomy, allegedly more household and childcare responsibilities) experienced more emotional exhaustion than those with more resources. Furthermore, its temporal focus, that is, its prediction that fewer resources predict later losses, proved especially useful in the context of the pandemic: Indeed, we found that exhaustion of these individuals went up and only went down again as more resources became available when lockdown measures decreased. However, this was not the case for all of the resources under investigation: Beyond their respective main effects, co‐worker social support, job insecurity, and work‐privacy conflicts had no temporal effect. This finding indicates that future research could attempt to identify the relative relevance of the many resources that Hobfoll (2001) lists.

In sum, our study highlights the insights that can be gained from a theoretical time‐related framework that allows mapping measurements to other events, allowing trajectory hypotheses. Thus, we encourage researchers to integrate time in their theoretical models of occupational health towards more dynamic models (Shipp & Cole, 2015).

Finally, our findings that work from home arrangements are associated with particularly high levels of exhaustion for certain groups are at odds with the extant literature on voluntary work from home, which reports positive consequences of telework for well‐being (Gajendran & Harrison, 2007). This is probably due to the forced nature of work from home in the context of the pandemic.

### Practical implications

Our results provide possible starting points for interventions aimed at reducing the psychological side‐effects of the pandemic for the working population. A key finding of our study is that women's psychological well‐being is more affected by the pandemic than men's, which may be the result of a shift towards traditional gender roles during the COVID‐19 pandemic (Rudolph et al., 2020). We can derive three points from our results to improve the situation for women: First, government should consider how they can support working women with pre‐school children through government lockdown measures (e.g., specific care offers). Second, organisations should increase employees' job autonomy (e.g., with flexible working from home arrangements and by providing the corresponding technology such as laptops) as soon as possible when a crisis arises. This provides the opportunity to better balance private and work‐related requirements. Third, we need more societal efforts as well as support from organisations for a more balanced distribution of child care and housework among couples, because a high level of partner support decreases women's exhaustion.

Another key finding is the strong and general impact of demands from the work‐home interface on employees' exhaustion. Organisations can help employees by fostering a family‐friendly culture (e.g., organisational and supervisor support) to mitigate stressors and strains. Moreover, research on resilience suggests that individuals learn new strategies for managing work‐related and private requirements during the COVID‐19 pandemic, which can increase their specific self‐efficacy beliefs dealing with future crises (Rudolph et al., 2020).

### Limitations and outlook

Despite its contributions, our study is not without limitations. First, the sample is not representative, but constitutes a convenience sample. This precludes the interpretation of sample descriptives such as mean levels of exhaustion as representing the population. However, statistical inference about relationships can be inferred from non‐random samples as in the present case (Highhouse & Gillespie, 2009). Especially when associated with large *t*‐values, relationships (e.g., between self‐efficacy and exhaustion) are likely to generalise to the population. Furthermore, we focused exclusively on employees in Germany, and are thus unable to speak to the probable psychological health issues that individuals who lost their job or who live in countries that are more ill‐prepared for dealing with the pandemic face. Furthermore, given that data collection commenced during the height of the pandemic in April 2020, a pre‐pandemic baseline for exhaustion is missing in this study. Nevertheless, we believe that our study offers a unique contribution to understanding the psychological impact of the pandemic.

## CONCLUSION

Our study shows that women's psychological health is more strongly affected by the COVID‐19 pandemic than men's psychological health. Future research needs to investigate whether this is due to the pandemic reinforcing traditional gender roles or due to other reasons. If governments and policy makers want to design interventions for softening the psychological consequences of the pandemic, they need to target women specifically. Conceptual starting points for such interventions could build on the constructs that helped women cope in the context of our study: Expanding childcare facilities, work design enabling increased levels of autonomy, and egalitarian approaches to sharing the burdens in the household such that working women's partners support them to further extents.

## Supporting information


**Appendix** **S1**. Supporting Information
