# Supplementary material for: Employee psychological well‐being during the COVID‐19 pandemic in Germany: A longitudinal study of demands, resources, and exhaustion
Source: Int J Psychol. 2021 Feb 21;56(4):532–50. doi: 10.1002/ijop.12743 (PMC8013458; doi:10.1002/ijop.12743)
Supplement: Supplementary file 1 — Appendix S1. Supporting Information [file IJOP-56-532-s001.docx]

See online Appendix A1, A2, Table S1 and Table S2 at <https://doi.org/10.17605/OSF.IO/AT3MY>.
